# Unmasking Early Cardiac Fibrosis in Sarcoidosis: The Role of Plasma Aldosterone and Cardiac MRI

**DOI:** 10.3390/jcm15020650

**Published:** 2026-01-14

**Authors:** Elias Giallafos, Evangelos Oikonomou, Niki Lama, Spiros Katsanos, Lykourgos Kolilekas, Evaggelos Markozanes, Varvara Pantoleon, Kostas Zisimos, Ourania Katsarou, Panagiotis Theofilis, Gesthimani Seitaridi, Ioannis Ilias, Grigoris Stratakos, Nikos Kelekis, Effrosyni D. Manali, Spiros Papiris, Georgios Marinos, Konstantinos Tsioufis, Gerasimos Siasos

**Affiliations:** 1 1st Department of Pulmunology, “Sotiria” General Hospital of Athens for Chest Diseases, Medical School, National and Kapodistrian University of Athens, 11527 Athens, Greece; grstrat@otenet.gr; 2 Cardiac Unit, “Aeginition” Hospital, Medical School, National and Kapodistrian University of Athens, 11527 Athens, Greece; spikats@yahoo.gr (S.K.); zisimoskostas@gmail.com (K.Z.); gseit24@hotmail.com (G.S.); 3 3rd Department of Cardiology, “Sotiria” General Hospital of Athens for Chest Diseases, Medical School, National and Kapodistrian University of Athens, 11527 Athens, Greece; raniakatsarou@yahoo.gr (O.K.); marinosgiorgos@hotmail.com (G.M.); ger_sias@hotmail.com (G.S.); 4 2nd Department of Radiology, Medical School, National and Kapodistrian University of Athens, 11527 Athens, Greece; niklampatr@gmail.com (N.L.); pantoleonvar@yahoo.com (V.P.); nkelekis@yahoo.com (N.K.); 5 7th Respiratory Clinic, “Sotiria” General Hospital of Athens for Chest Diseases, 11527 Athens, Greece; lykol@yahoo.gr (L.K.); markozanes@yahoo.com (E.M.); 6 1st Department of Cardiology, “Hippocration” General Hospital of Athens, Medical School, National and Kapodistrian University of Athens, 11527 Athens, Greece; panos.theofilis@hotmail.com (P.T.); ktsioufis@gmail.com (K.T.); 7 Endocrinology Unit, “Hippocration” General Hospital of Athens, 11527 Athens, Greece; iiliasmd@yahoo.com; 8 2nd Department of Pulmunology, Attikon Hospital, Medical School, National and Kapodistrian University of Athens, 11527 Athens, Greece; fmanali@otenet.gr (E.D.M.); papiris@otenet.gr (S.P.)

**Keywords:** cardiac sarcoidosis, aldosterone, fibrosis, inflammation

## Abstract

**Background/Objectives:** Cardiac sarcoidosis (CS) is a challenging diagnosis due to its subclinical progression and the limitations of existing screening tools. Cardiac magnetic resonance (CMR) and PET/CT imaging have improved diagnosis and detection. Aldosterone, a hormone with known profibrotic effects, may offer additional diagnostic value. We therefore aimed to determine whether plasma aldosterone level is associated with myocardial fibrosis, independent of active inflammation, in CS. **Methods:** This observational study included 541 patients with biopsy-proven sarcoidosis and preserved left ventricular ejection fraction (LVEF ≥ 50%). All underwent CMR with extracellular volume (ECV) mapping and 18F-FDG PET/CT to assess myocardial fibrosis and inflammation, respectively. Plasma aldosterone levels were also measured. **Results:** Plasma aldosterone levels were significantly higher in patients with cardiac sarcoidosis (172 [IQR 106–235] pg/mL) compared to those without cardiac involvement (143 [100–205] pg/mL, *p* = 0.02). Aldosterone was independently associated with the presence of late gadolinium enhancement (LGE) on CMR (OR 1.002 per 1 pg/mL increase; 95% CI 1.001–1.004, *p* = 0.04) and with higher ECV values (β = 0.008 per 1 pg/mL, *p* = 0.001). Regression analysis showed that aldosterone is associated with ECV (b-0.009, CI: 0.002–0.016, *p* = 0.009) and there was no interaction according to LGE status indicating a relationship with diffuse myocardial fibrosis even in the absence of visible scarring. No association was observed with T1-, T2-, or PET/CT-defined inflammation. **Conclusions:** Plasma aldosterone is a robust marker of myocardial fibrosis in sarcoidosis, particularly in early or subclinical stages. Its correlation with ECV—but not with inflammatory imaging markers—suggests its link with myocardial diffuse fibrotic remodeling before, and independently of, overt scarring or inflammation.

## 1. Introduction

Sarcoidosis is a systemic granulomatous disease of unknown etiology, with frequent involvement of the lungs and lymphatic system. Cardiac sarcoidosis (CS), although less commonly diagnosed, is a critical manifestation due to its potential for sudden cardiac death, arrhythmias, conduction disturbances, and heart failure. Early detection of cardiac involvement remains challenging due to the heterogeneity of presentations and limitations of existing diagnostic criteria [1,2].

Recent advancements in cardiac imaging, including cardiac magnetic resonance (CMR) imaging with extracellular volume (ECV) mapping, late gadolinium enhancement (LGE), T1 and T2 mapping, and positron emission tomography/computed tomography (PET/CT), have improved the sensitivity of detecting subclinical myocardial involvement in sarcoidosis [2,3]. However, the identification of robust biomarkers to complement imaging and improve diagnostic specificity and risk stratification is still pending.

Aldosterone, a mineralocorticoid hormone, through its profibrotic and pro-inflammatory effects, has been implicated in myocardial fibrosis and remodeling [4]. Fibrosis is a feature of sarcoidosis, playing a key role in both its clinical course and underlying pathophysiology. Inflammatory myocardial involvement may precede myocardial fibrosis, while progressive pulmonary fibrosis contributes to the development of sarcoidosis-associated pulmonary hypertension [3]. Importantly, extensive replacement fibrosis of the myocardium may cause either atrioventricular conduction abnormalities or heart failure with impaired systolic and or diastolic left ventricular function [5]. But beyond granulomatous inflammation, the renin–angiotensin–aldosterone system may regulate the inflammatory pathway and contribute to related inflammation [6].

Elevated serum aldosterone levels have been associated with adverse cardiac outcomes and heart failure morbidity [7]. Moreover, in sarcoidosis, aldosterone may reflect a parallel fibrotic axis distinct from inflammation and caseating granulomas and, in ex vivo models of sarcoidosis granulomas, mineralocorticoid receptor inhibitors attenuate pro-inflammatory cytokine release [6]. Despite its known role in hypertension, chronic kidney disease, and heart failure, its potential value as a diagnostic or prognostic marker in sarcoidosis, particularly in those without overt cardiac dysfunction, has not been well explored. There are also no data on how aldosterone levels are implicated in the course of CS, from preclinical stages to overt symptomatic heart disease, or on how inhibition of mineralocorticoid action may beneficially affect disease activity.

Therefore, we examine the association between circulating aldosterone levels and CMR markers of cardiac involvement in patients with a new onset diagnosis of sarcoidosis.

## 2. Materials and Methods

### 2.1. Study Population

Consecutive adult patients with biopsy-proven sarcoidosis referred to our tertiary care center for evaluation of cardiac involvement were enrolled in this observational study. Patients were excluded from the study if they met any of the following criteria:

1. Left ventricular ejection fraction (LVEF) less than 50% on echocardiography.
2. Evidence of New York Heart Association (NYHA) class III–IV heart failure, ischemic heart disease, significant valvular disease, congenital heart disease, or other non-sarcoid structural cardiomyopathies that could confound cardiac imaging or biomarker interpretation.
3. Patients on mineralocorticoid receptor antagonist treatment.

Demographic and clinical data as well as medication history were recorded at enrollment, including age, sex, smoking, body mass index (BMI), comorbidities, immunosuppressive therapy, and chest radiographic disease stage according to Scadding criteria [8].

The diagnosis of cardiac sarcoidosis was based on the combination of the Japanese Circulation Society’s 2016 Guideline on Diagnosis and Treatment of Cardiac Sarcoidosis and on biopsy-proven sarcoidosis in organs other than the heart [9].

All participants provided written informed consent prior to their inclusion in the study. The study protocol was approved by the Ethics Committee of “Sotiria” General Hospital of Chest Diseases (4393, 8 May 2017), and all procedures were conducted in accordance with the Declaration of Helsinki and relevant institutional guidelines.

### 2.2. Cardiac Imaging

#### 2.2.1. Cardiac Magnetic Resonance

All patients underwent a CMR examination using a 3.0T MRI Philips Achieva (Philips Medical Systems, Best, The Netherlands) scanner. The imaging protocol included cine imaging using a retrospective ECG-gated balanced turbo field echo bTFE to assess LV and right ventricular (RV) function, mass, and dimensions (TE/TR = 1.5/3 ms, slice thickness = 8 mm without gap, flip angle = 40°, acquisition matrix 185 × 183); an ECG-triggered black blood T2 STIR to assess myocardial edema (TE/TR = 75 ms/2 RR intervals, slice thickness = 10 mm, gap = 1 mm, flip angle = 90°, inversion time delay = 210 ms, acquisition matrix 200 × 141); an ECG-triggered phase-sensitive inversion (TE/TR = 3.0/6.1 ms, acquisition matrix 200 × 148, slice thickness = 10 mm with gap = 2 mm, IR value selected after Look-Locker acquisition) 10 min after the gadolinium injection to assess myocardium viability; a 2D retrospective ECG-gated phase-contrast sequence to quantify the flow of the aorta and pulmonary vessel (TE/TR = 4.8/2.8 ms, flip angle = 10°, slice thickness = 8 mm). Native and late gadolinium-enhanced T1 maps were also acquired using a modified Look-Locker Inversion recovery sequence to estimate structural lesions (TE/TR = 1.03/2.2, slice thickness = 10 mm with gap = 10 mm, acquisition matrix 120 × 180). Image post-processing was performed using commercially available clinical CMR software cvi 42 version (Circle Cardiovascular Imaging, Calgary, AB, Canada). All CMR images were analyzed by the same two experienced imaging readers, blinded to clinical data, with discrepancies resolved by consensus.

CMR protocols adhered to current consensus guidelines and included the following components:

- T1 and T2 Mapping: Native T1 and T2 values were obtained to assess diffuse myocardial fibrosis and edema [10]. Upper reference values for our laboratory for T1 and T2 are 1290 ms and 54 ms, respectively.
- LGE: LGE imaging was performed 10–15 min after intravenous administration of gadolinium-based contrast (0.1 mmol/kg), using inversion recovery sequences optimized to null normal myocardium. The presence, pattern, and extent of LGE were assessed visually and semi-quantitatively. LGE was considered positive if non-ischemic patterns of enhancement (mid-wall, patchy, or subepicardial) were observed, consistent with granulomatous inflammation or fibrosis.
- ECV Quantification: ECV was calculated using pre- and post-contrast T1 mapping values and contemporaneous hematocrit levels, allowing for quantification of myocardial matrix expansion. ECV measurements were global, using 3 different short-axis slices in the diastolic phase post-processed by the dedicated software. T1-post images were acquired at 15 min post-contrast. Major areas of LGE were excluded from measurements in accordance with the needs of this research, as well as areas of major artifacts. A qualified Medical Physicist was responsible for the quality validation of parametric mapping techniques. ECV was reported as a percentage of total myocardial volume [10]. The upper reference value for our laboratory for ECV is 29%.

#### 2.2.2. PET/CT

Scanning with 18F-fluorodeoxyglucose PET/CT (18F-FDG PET/CT) was applied to detect active myocardial inflammation. A standardized patient preparation protocol was used to suppress physiological myocardial glucose uptake, consisting of a high-fat, low-carbohydrate diet for 24 h followed by a 12 h fasting period. Intravenous administration of 18F-FDG was followed by PET/CT image acquisition approximately 60 min post-injection. PET/CT scans were evaluated for the presence and distribution of myocardial FDG uptake. A positive cardiac PET/CT study was defined by focal or focal-on-diffuse patterns of myocardial FDG uptake, considered indicative of active inflammation. A negative cardiac PET/CT was defined as the absence of pathological myocardial FDG uptake, typically characterized by no uptake or a diffusely low/absent signal, indicating no evidence of active myocardial inflammation.

All imaging studies (CMR and PET/CT) were interpreted independently by two experienced cardiovascular imaging specialists, who were blinded to the clinical, biochemical, and histopathological data.

### 2.3. Biomarker Assessment

Peripheral venous blood samples were obtained from each participant under fasting conditions. All blood samples were collected from fasting morning samples, collected after participants had been ambulant (upright) for 1 h.

As per protocol, none of the patients were receiving mineralocorticoid receptor antagonist therapy or corticosteroids. All patients were instructed to discontinue diuretics for at least 4 weeks and angiotensin-converting enzyme inhibitors, angiotensin receptor blockers, or b-blockers for at least 2 weeks prior to aldosterone measurement. Arterial hypertension was managed with non-dihydropyridine calcium channel blockers. Participants were instructed not to modify their habitual dietary sodium intake during the study period.

Plasma aldosterone concentration (PAC) and Brain Natriuretic Peptide (BNP) were quantified. Plasma renin activity (PRA) was determined using a radioimmunoassay method and expressed as ng/mL/h. The aldosterone-to-renin ratio (ARR) was calculated as PAC (ng/dL) divided by plasma renin activity (ng/mL/h). For ARR calculation, aldosterone concentrations measured in pg/mL were converted to ng/dL by dividing by 10. An ARR value greater than 30 (ng/dL per ng/mL/h) was considered significant.

Additional laboratory tests obtained at the time of sampling included renal function, C-reactive protein (CRP), cardiac troponin I, and serum angiotensin-converting enzyme. All samples were aliquoted and stored at −80 °C in a biorepository until batch analysis to minimize inter-assay variability.

### 2.4. Statistical Analysis

Continuous variables were tested for normality of distribution with the Kolmogorov–Smirnov test. Not normally distributed variables were logarithmically transformed and, after confirmation of normality, were analyzed using parametric tests (*t*-test and Pearson correlation). Normally distributed variables were expressed as mean ± SD or as median with interquartile range for variables that were initially not normally distributed, regardless of logarithmic transformation. Categorical variables were presented as percentages.

Comparisons between patients with CS and without cardiac involvement (Non-CS) were conducted using independent sample *t*-tests for continuous variables. The Chi-square test was used for comparison of categorical variables. Pearson’s correlation coefficient was applied to test possible associations between continuous variables (e.g., plasma aldosterone and cardiac MRI parameters).

To further assess the relationship between plasma aldosterone levels and cardiac imaging findings, regression analyses were performed. Binary logistic regression was used to determine whether plasma aldosterone levels were independently associated with the presence of LGE on CMR imaging. Linear regression was used to evaluate the association between aldosterone levels and extracellular volume (ECV). The model was adjusted for potential confounders. To assess effect modification by LGE status (positive or negative), an interaction term was created as the product of LGE status and aldosterone levels and was entered in the multivariable regression model together with the corresponding main effects. A two-sided *p*-value < 0.05 was considered statistically significant. All statistical calculations were performed with SPSS version 23.0 (SPSS Inc., Chicago, IL, USA).

## 3. Results

### 3.1. Study Population Characteristics

A total of 541 patients with sarcoidosis were included in the study. Participants were categorized according to the Scadding radiographic staging criteria: Stage I (*n* = 287), Stage II (*n* = 195), Stage III (*n* = 43), and Stage IV (*n* = 16). From the study population, 132 (22.5%) were diagnosed with CS, while 409 (69.8%) had no clinical or imaging evidence of cardiac involvement (Non-CS) (Table 1).

Patients with CS were older (55 ± 12 years vs. 52 ± 11 years, *p* = 0.007) and more frequently male (50% vs. 37%, *p* = 0.006) compared to those with Non-CS. No significant difference was observed in smoking status or the prevalence of diabetes mellitus. However, hypertension was more prevalent (29.1% vs. 18.3%, *p* = 0.008) and BMI was slightly higher (28.8 ± 5.5 vs. 27.8 ± 4.7 kg/m^2^, *p* = 0.04) in the CS compared to the Non-CS group. CRP did not differ significantly, but serum creatinine levels were marginally elevated in CS compared to Non-CS patients (0.87 ± 0.28 mg/dL vs. 0.80 ± 0.22 mg/dL, *p* = 0.002).

Regarding cardiac imaging, CS exhibited a significantly increased T1 time (1266 ± 36 ms vs. 1256 ± 40 ms, *p* = 0.045), T2 time (50 ± 5 ms vs. 48 ± 3 ms, *p* = 0.001), and ECV (29 ± 3% vs. 27 ± 3%, *p* < 0.001) and reduced LVEF (59 ± 8% vs. 62 ± 5%, *p* < 0.001) compared to Non-CS patients.

Plasma BNP and troponin levels were also higher in the CS compared to the Non-CS group (BNP 35 (14, 85) pg/mL vs. 23 (10, 55) pg/mL, *p* = 0.001; troponin 2.70 (1.10, 6.30) pg/mL vs. 1.30 (0.50, 2.50) pg/mL, *p* < 0.001). Importantly, aldosterone levels were also higher in CS compared to Non-CS patients (172 (106–235) pg/mL vs. (143 (100–205) pg/mL, *p* = 0.02). There was no difference in pulmonary arterial systolic pressure (PASP) between CS and Non-CS patients.

### 3.2. Association of Plasma Aldosterone with Imaging Findings in CS

**Figure 1:**
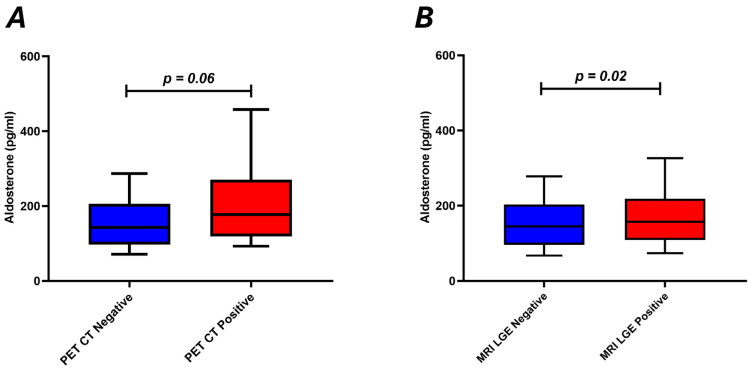
Boxplots showing aldosterone levels in patients with sarcoidosis according to positive or negative cardiac PET CT (**A**) and according to positive or negative LGE cardiac MRI (**B**).

We next proceeded to the evaluation of the association of plasma aldosterone levels with cardiac PET/CT findings (positive or negative) and with evidence of CMR involvement (ECV, T1, T2, and LGE findings). There was no difference in plasma aldosterone levels between patients with negative or positive PET/CT [143 (98, 205) pg/mL vs. 177 (120–270) pg/mL, *p* = 0.06] (Figure 1A). Plasma aldosterone levels were higher in patients with positive LGE in CMR compared to negative LGE [158 (109–219) pg/mL vs. 145 (96, 203) pg/mL, *p* = 0.03] (Figure 1B).

**Figure 2:**
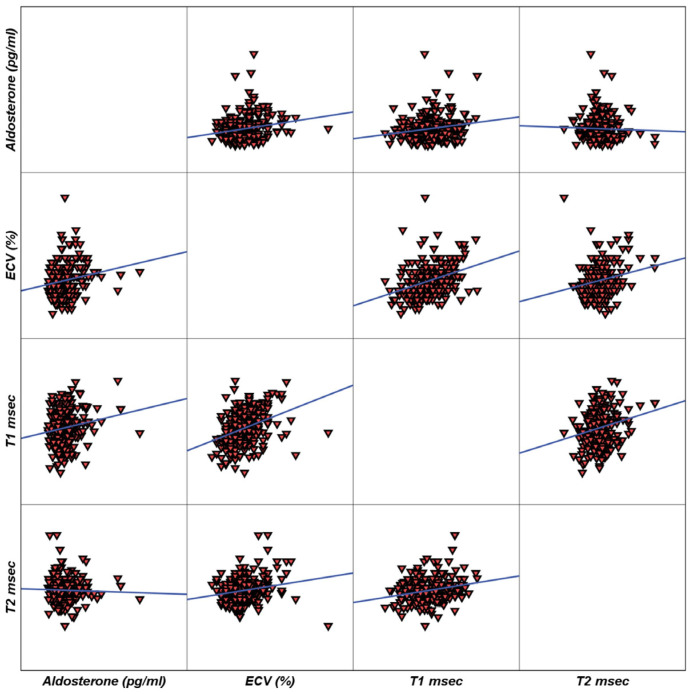
Correlogram showing the association of aldosterone levels with ECV, T1, and T2.

Moreover, in the study population, there was a significant correlation of plasma aldosterone levels with ECV (r = 0.18, *p* = 0.002), while there is no association with either T1 (r = 0.10, *p* = 0.07) nor with T2 (r = −0.04, *p* = 0.44) (Figure 2).

To explore if the associations of plasma aldosterone levels with LGE or ECV were independent of possible confounders (age, sex, BMI, hypertension, CRP, creatinine, BNP, Troponin I, and LVEF), we proceeded to regression analyses. Accordingly, we noted that for every 100 pg/mL increase in plasma aldosterone levels, the odds of having positive LGE increased by 20% (Exp(B) = 1.002; 95% CI: 1.001–1.004; *p* = 0.04) (Table 2). Moreover, for every 100 pg/mL increase in plasma aldosterone levels, there was an almost 1% increase in ECV (b = 0.009, 95% CI: 0.002–0.016, *p* = 0.009) (Table 3). No significant interaction was observed between aldosterone levels and LGE status, indicating that the association between aldosterone and ECV was present even in subjects without LGE.

## 4. Discussion

In this observational study of patients with biopsy-proven sarcoidosis and preserved LVEF, we found that aldosterone levels were higher in patients with CS compared with those without CS, despite no difference in the ARR. We also observed that circulating plasma aldosterone levels were independently associated with myocardial ECV, a CMR-derived marker of diffuse myocardial fibrosis. This association persisted after adjustment for traditional clinical and biochemical confounders, including age, sex, hypertension, renal function, BNP, and troponin. Moreover, aldosterone levels were not associated with PET/CT findings nor with T1 or T2 mapping values derived from CMR. In addition, the association between aldosterone and ECV was independent of LGE even in LGE-negative cases, a subgroup typically considered to lack overt myocardial scarring, suggesting a potential role for aldosterone as a marker of early subclinical, diffuse interstitial fibrotic remodeling.

CMR imaging plays a pivotal role in the diagnosis of CS, allowing for non-invasive detection of both focal and diffuse myocardial pathology. While LGE is a well-established marker of replacement fibrosis and is incorporated into diagnostic algorithms for CS [3,11], its sensitivity for early or diffuse interstitial disease is limited. In contrast, ECV mapping provides a quantitative assessment of myocardial matrix expansion, making it particularly valuable for identifying preclinical or subtle fibrotic changes [12]. Moreover, CMR-derived T1 and T2 mapping values are considered sensitive to acute inflammatory changes and myocardial edema [13] and in the setting of CS, they can be used to help in the identification of acute inflammatory changes and focal or diffuse myocardial edema with increased intracellular and/or extracellular water [14]. Additionally, PET/CT, by identifying active myocardial inflammation through focal pathologic glucose uptake, plays a central role in CS and may identify cases with negative CMR findings [15].

The lack of association between aldosterone levels and PET/CT and CMR-derived T1 or T2 values further suggests the possibility that aldosterone marks a distinct fibrotic process [4] in contrast to inflammatory biomarkers or imaging findings, which fluctuate with disease activity but may not predict incident fibrosis or remodeling.

Furthermore, there was no interaction between LGE status (negative or positive) or plasma aldosterone levels with ECV. This finding further highlights that aldosterone may be linked specifically to early, diffuse fibrosis rather than to late-stage scar formation. This distinction is clinically important as it implies that aldosterone elevation may precede irreversible structural damage and that elevated aldosterone in LGE-negative patients may indicate a fibrotic trajectory that has not yet been detected with conventional imaging markers. Based on our study, we cannot determine whether elevations in aldosterone precede ECV expansion. However, from an etiological and temporal perspective, it is plausible that aldosterone elevation precedes ECV expansion, a notion supported by the lack of association between aldosterone levels and CMR T1 and T2 indices of active inflammation and myocardial edema.

Another relevant consideration is whether aldosterone excess in CS reflects systemic neurohormonal activation or local myocardial synthesis. The data presented in this manuscript do not allow for definitive conclusions regarding the source of elevated plasma aldosterone levels. However, given that aldosterone was assessed using plasma measurements rather than regional myocardial sampling, these findings are consistent with a predominantly systemic contribution. Nevertheless, prior clinical and experimental studies have linked sarcoidosis to increased angiotensin-converting enzyme activity within granulomatous tissues. In addition, macrophages involved in sarcoid granuloma formation are regulated by the renin–angiotensin–aldosterone system and may produce aldosterone locally, thereby activating mineralocorticoid receptors within the inflammatory–fibrotic myocardial milieu [6]. Regarding our findings, the lack of a significant difference in ARR suggests that aldosterone elevation may occur independently of renin–angiotensin system activation, supporting an alternative inflammatory pathway.

Several clinical implications may arise based on the findings of our study. First, they suggest that aldosterone may serve as an early marker of myocardial remodeling in sarcoidosis, identifying patients at risk of developing fibrotic cardiac involvement even when replacement fibrosis is absent. This is particularly valuable in clinical settings where early detection of CS remains challenging and where standard imaging findings are considered negative for myocardial involvement.

Second, because aldosterone can be pharmacologically modulated and has a well-established role in myocardial fibrosis, our findings provide a rationale for therapeutic intervention in this population. These findings generate the hypothesis that mineralocorticoid receptor antagonists, such as spironolactone and eplerenone, or newer non-steroidal agents may be beneficial in patients with sarcoidosis, even in the presence of normal LVEF and the absence of overt heart failure. Such approaches have been shown to attenuate fibrotic remodeling and improve clinical outcomes in other cardiac conditions [16,17]. In line with this evidence, mineralocorticoid receptor antagonists may represent a promising therapeutic strategy in CS, particularly among patients with elevated aldosterone levels and early fibrotic changes. Contemporary sarcoidosis therapy addresses two complementary therapeutic targets: active inflammation and established organ fibrosis. Active disease is managed with immunomodulatory treatments, such as corticosteroids, azathioprine, or other immunosuppressive agents. In contrast, established organ fibrosis is addressed through management of complications resulting from fibrotic damage. Aldosterone measurement, as a low-cost, accessible, and noninvasive test, may serve as a complementary tool for longitudinal monitoring of fibrotic risk or therapeutic response. This approach may be particularly useful in patients with equivocal or borderline imaging findings.

Collectively, these findings suggest that aldosterone may play a role in the evaluation and management of CS, although further studies are needed to confirm its additive clinical value.

## 5. Conclusions

In patients with cardiac sarcoidosis and preserved left ventricular ejection fraction, plasma aldosterone levels are independently associated with myocardial extracellular volume, reflecting diffuse fibrosis. This is evident even in patients without overt myocardial scarring on cardiac magnetic resonance imaging, suggesting that aldosterone may additionally act as a biomarker of early diffuse fibrotic remodeling, preceding the development of focal replacement fibrosis. These findings support further investigation of aldosterone as a diagnostic, prognostic, and potentially therapeutic target in cardiac sarcoidosis.

## Figures and Tables

**Table 1:** Demographic, clinical, laboratory, and imaging characteristics of the study population according to cardiac involvement.

	Study Population	CS	Non-CS	*p*-Value
	541	132	409	
Age (years)	53 ± 12	55 ± 12	52 ± 11	0.007
Male Sex (%)	39	50	37	0.006
Smoking (%)	17	16	17	0.06
BMI (kg/m^2^)	28.22 ± 5.17	28.79 ± 5.51	27.76 ± 4.73	0.04
Diabetes Mellitus (%)	11	15	10	0.09
Hypertension (%)	21	29.1	18.3	0.008
CRP (U/L)	0.29 (0.12, 0.57)	0.28 (0.12, 0.53)	0.29 (0.12, 0.55)	0.41
Cre (mg/dL)	0.81 ± 0.23	0.87 ± 0.28	0.80 ± 0.22	0.002
T1 (ms)	1259 ± 39	1266 ± 36	1256 ± 40	0.045
T2 (ms)	49 ± 4	50 ± 5	48 ± 3	0.001
ECV (%)	27 ± 3	29 ± 3	27 ± 3	<0.001
LVEF (%)	61 ± 6	59 ± 8	62 ± 5	<0.001
BNP (pg/mL)	25 (11, 60)	35 (14, 85)	23 (10, 55)	0.001
SACE U/L	50 ± 37	48 ± 33	51 ± 38	
Troponin (pg/mL)	1.45 (0.60, 3.13)	2.70 (1.10, 6.30)	1.30 (0.50, 2.50)	<0.001
Aldosterone (pg/mL)	149 (100, 212)	172 (106, 235)	143 (100, 205)	0.02
ARR (ng/dL per ng/mL/h)	8.6 (5.0, 15.4)	9.7 (5.0, 19.1)	8.7 (5.2, 15.1)	0.38
High ARR (%)	8	11	6	0.16
PASP (mmHg)	26 ± 8	27 ± 9	26 ± 7	0.21

CS: cardiac sarcoidosis; BMI: body mass index; CRP: C-reactive protein; ECV: extra cellular volume; LVEF: left ventricular ejection fraction; BNP: b-type natriuretic peptide; SACE: serum angiotensin-converting enzyme; ARR: aldosterone-to-renin ratio; PASP: pulmonary artery systolic pressure.

**Table 2:** Binary logistic regression with positive LGE as dependent variable.

	Exp(B)	95% CI		*p*-Value
		Lower	Higher	
Age (years)	1.021	0.998	1.045	0.08
Male sex	1.029	0.637	1.663	0.08
BMI (kg/m^2^)	1.005	0.961	1.051	0.83
Arterial hypertension	0.910	0.498	1.663	0.76
Creatine (mg/dL)	0.949	0.703	1.280	0.73
BNP (pg/mL)	1.004	1.001	1.008	0.02
Troponin (pg/mL)	0.983	0.957	1.010	0.22
LVEF (%)	0.968	0.918	1.021	0.24
Aldosterone (pg/mL)	1.002	1.001	1.004	0.04

LGE: late gadolinium enhancement; BMI: body mass index; BNP: B-type natriuretic peptide; LVEF: left ventricular ejection fraction. Model performance summary: χ^2^ = 22.908, Nagelkerke R^2^ = 0.085, overall accuracy = 62.8%.

**Table 3:** Regression analysis with ECV as dependent variable.

	b	95% CI		*p*-Value
		Lower	Higher	
Age (years)	0.009	−0.041	0.058	0.73
Male sex	−0.556	−1.847	0.735	0.40
BMI (kg/m^2^)	−0.069	−0.168	0.031	0.17
Arterial hypertension	0.420	−0.945	1.784	0.55
Creatine (mg/dL)	−2.899	−6.301	0.504	0.09
BNP (pg/mL)	−0.002	−0.009	0.005	0.54
Troponin (pg/mL)	0.015	−0.035	0.064	0.56
LVEF (%)	−0.028	−0.145	0.089	0.64
Aldosterone (pg/mL)	0.009	0.002	0.016	0.009
LGE positive	0.804	−0.170	1.779	0.11
Interaction LGE × aldosterone	−0.004	−0.013	0.006	0.45

LGE: late gadolinium enhancement; BMI: body mass index; BNP: B-type natriuretic peptide; LVEF: left ventricular ejection fraction. Model performance summary: adjusted R^2^ = 0.021.

## Data Availability

The datasets supporting the findings of this research are available from the corresponding author upon reasonable request.

## Abbreviations

The following abbreviations are used in this manuscript:

CS	Cardiac sarcoidosis
CMR	Cardiac magnetic resonance
ECV	Extracellular volume
LGE	Late gadolinium enhancement
PET/CT	Positron emission tomography/computed tomography
LVEF	Left ventricular ejection fraction
NYHA	New York Heart Association
BMI	Body mass index
RV	Right ventricular
PAC	Plasma aldosterone concentration
BNP	Brain natriuretic peptide
ARR	Aldosterone-to-renin ratio
CRP	C-reactive protein
SACE	Serum angiotensin-converting enzyme
PASP	Pulmonary artery systolic pressure
